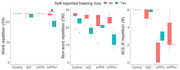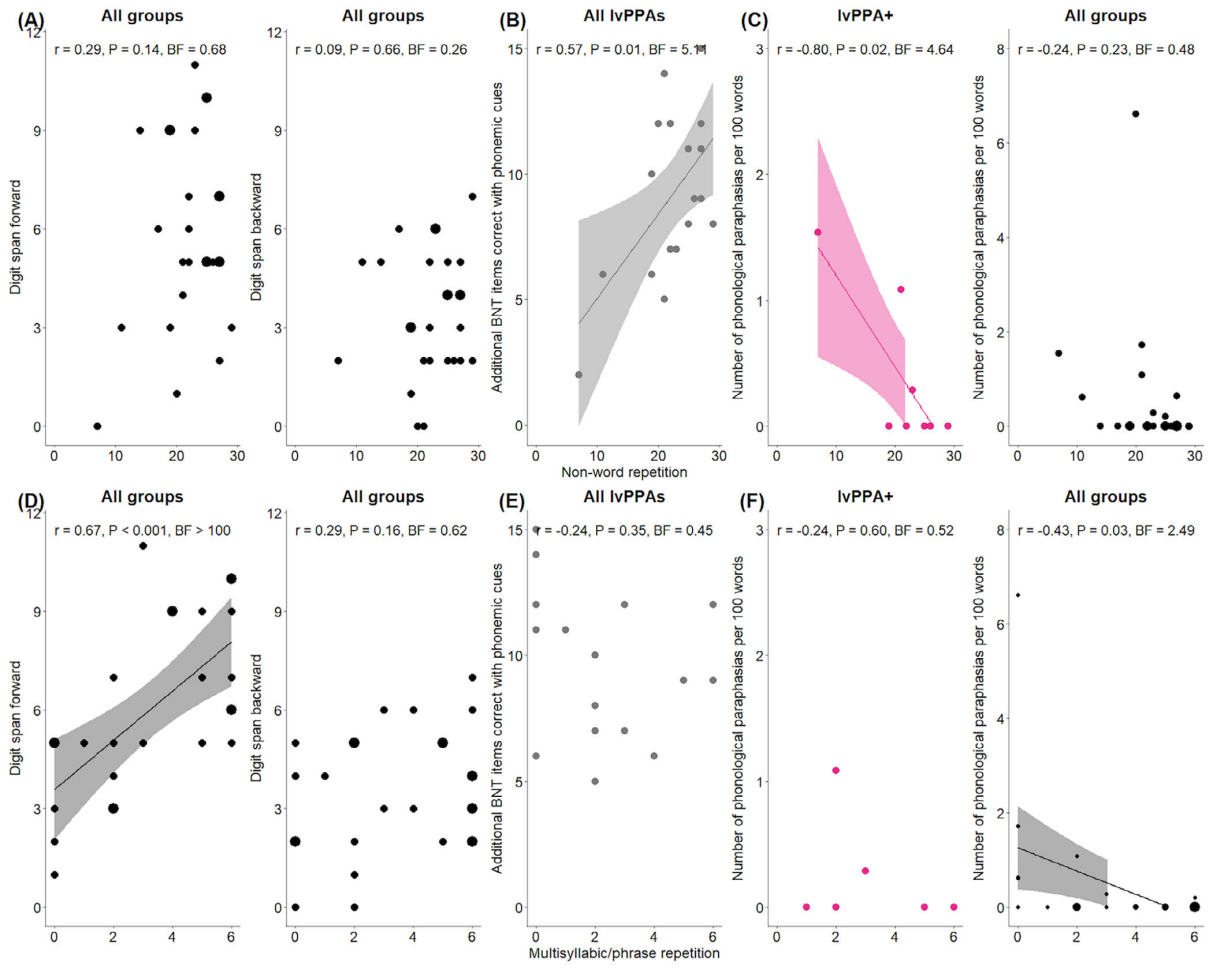# Disentangling phonology from phonological short‐term memory in the logopenic variant of primary progressive aphasia and typical, amnestic Alzheimer's disease

**DOI:** 10.1002/alz70857_103483

**Published:** 2025-12-25

**Authors:** Shalom K Henderson, Ajay D Halai, Kamen A Tsvetanov, Thomas E Cope, Karalyn E Patterson, James B Rowe, Matthew A Lambon Ralph

**Affiliations:** ^1^ University of Cambridge, Cambridge, United Kingdom; ^2^ Department of Clinical Neurosciences, University of Cambridge, Cambridge, Cambridgeshire, United Kingdom; ^3^ University of Cambridge, Cambridge, ‐, United Kingdom

## Abstract

**Background:**

Impaired phonological short‐term memory is a core feature of the logopenic variant of primary progressive aphasia (lvPPA), but it is not clear whether a core phonological processing deficit is also present. We asked three questions: (1) beyond short‐term memory impairment, do people with lvPPA have an impairment within phonology itself?; (2) is their performance in working memory and confrontation naming reflective of this phonological impairment and/or other key contributing deficits?; and (3) is their repetition performance related to structural and functional differences in key language‐dominant regions?

**Method:**

We compared non‐word and word repetition and short‐term memory performance in patients with typical, amnestic Alzheimer's disease (tAD, *n* = 9), lvPPA per consensus criteria (*n* = 10), and others who previously satisfied definitions of lvPPA but had progressed with multi‐domain cognitive impairments (lvPPA+, *n* = 8).

**Result:**

Bayesian analyses revealed no group differences in phonological tasks of word and non‐word repetition. We found very strong evidence for an effect of self‐reported hearing loss on both word and non‐word repetition, but not multi‐syllabic word/phrase repetition. A comparison of phonological (as indexed by non‐word repetition) *versus* working memory (as measured by multisyllabic word repetition and digit span) and confrontation naming tasks produced either no evidence or evidence for no correlation. Confrontation naming correlated positively with multisyllabic word/phrase repetition and semantic assessments across the whole group. Beyond the expected grey matter reductions in patients relative to controls in the left posterior superior temporal gyrus, anterior temporal lobe, and inferior temporal gyrus, the only anecdotal evidence in patients was for an association between non‐word repetition and functional connectivity between dorsal premotor and posterior superior temporal gyrus after controlling for the grey matter volumes in these regions.

**Conclusion:**

We have shown that deficits in “pure” phonological tasks, namely single word and non‐word repetition, are (i) not dependent on working memory and (ii) greater in patients with a self‐reported hearing loss across all groups with lvPPA or tAD. Our results suggest that instead of having a core phonological impairment, lvPPA patients have a working memory/buffering impairment that adversely affects their performance on length‐dependent working memory tasks.